# Restoring Somatosensation: Advantages and Current Limitations of Targeting the Brainstem Dorsal Column Nuclei Complex

**DOI:** 10.3389/fnins.2020.00156

**Published:** 2020-02-28

**Authors:** Alastair J. Loutit, Jason R. Potas

**Affiliations:** School of Medical Sciences, UNSW Sydney, Sydney, NSW, Australia

**Keywords:** neural coding, brain-machine interface, neuroprosthesis, cuneate, gracile, tactile, proprioception, sensory feedback

## Abstract

Current neural prostheses can restore limb movement to tetraplegic patients by translating brain signals coding movements to control a variety of actuators. Fast and accurate somatosensory feedback is essential for normal movement, particularly dexterous tasks, but is currently lacking in motor neural prostheses. Attempts to restore somatosensory feedback have largely focused on cortical stimulation which, thus far, have succeeded in eliciting minimal naturalistic sensations. Yet, a question that deserves more attention is whether the cortex is the best place to activate the central nervous system to restore somatosensation. Here, we propose that the brainstem dorsal column nuclei are an ideal alternative target to restore somatosensation. We review some of the recent literature investigating the dorsal column nuclei functional organization and neurophysiology and highlight some of the advantages and limitations of the dorsal column nuclei as a future neural prosthetic target. Recent evidence supports the dorsal column nuclei as a potential neural prosthetic target, but also identifies several gaps in our knowledge as well as potential limitations which need to be addressed before such a goal can become reality.

## Introduction

A current challenge in neural prosthetic development is how to artificially activate the central nervous system to restore touch and proprioceptive sensation to tetraplegic patients (Lebedev and Nicolelis, 2017). Developments in the neural prosthetics field have raised the possibility of restoring limb movement, either by functional electrical stimulation of a tetraplegic patient’s own muscles (Ajiboye et al., 2017) or by facilitating control of a robotic limb (Collinger et al., 2013). In one paradigm, brain signals coding a patient’s intended movement can be acquired and decoded to control a robotic limb via thought alone. Improvements in decoding algorithms and anthropomorphic robotic limb design have enabled complex, thought-controlled movements, but realistic limb movement will require restored somatosensation to facilitate closed-loop feedback control (O’Doherty et al., 2011; Delhaye et al., 2016).

Recently, human intracortical microstimulation (ICMS) has been successful in eliciting minimal naturalistic tactile and proprioceptive sensations (Flesher et al., 2016; Salas et al., 2018). In one subject, some ICMS protocols targeted in somatosensory cortex were perceived as natural sensations such as squeezing, taps, vibration, and directional arm movement (Salas et al., 2018), whereas in another subject they were perceived as paraesthesia, buzzing, or almost natural (Flesher et al., 2016). Studies in monkeys have shown that different cortical stimulation parameters can elicit perception of variations in pressure, stimulus location, and virtual textures (Tabot et al., 2013, 2015; Kim et al., 2015), and can be used to provide artificial somatosensory feedback for movement control (O’Doherty et al., 2009; Klaes et al., 2014; O’Doherty et al., 2019). While these advances are promising, the effective restoration of natural tactile and proprioceptive feedback still faces many challenges.

One aspect requiring further investigation is whether other targets on the somatosensory neuraxis might offer advantages over the cortex for restoring somatosensory function. The complexity of neural networks in the cortex makes it a difficult region in which to target microstimulation. There has been better success in restoring somatosensory percepts in amputees by interfacing with peripheral nerves where the labeled line arrangement of afferent fibers has led to effective artificial recreation of somatosensory signals (Clark et al., 2014; Tan et al., 2015; Oddo et al., 2016; Valle et al., 2018; George et al., 2019). Users of some state-of-the-art peripheral nerve interfaces that used biomimetic stimulation approaches report that they feel as if they are grasping a real object and they can feel the intensity of the grasping force applied by the robotic hand (Valle et al., 2018). Another subject was able to determine whether the robotic arm held a golf ball or a lacrosse ball, based on their size, and discriminated the compliance of a soft foam block and hard plastic block during active manipulation with a robotic arm (George et al., 2019). The speed with which the subject could discriminate in these two tasks was significantly increased with biomimetic feedback algorithms, compared to simpler feedback algorithms using linear signal amplitude or frequency changes associated with the sensor output. Current peripheral neural prostheses outperform cortical ones for sensorimotor tasks. While integrating somatosensory feedback through ICMS is an impressive recent feat in humans, the subject still used a combination of visual and somatosensory feedback, and trained on the task for 2 years (Flesher et al., 2019).

**FIGURE 1:**
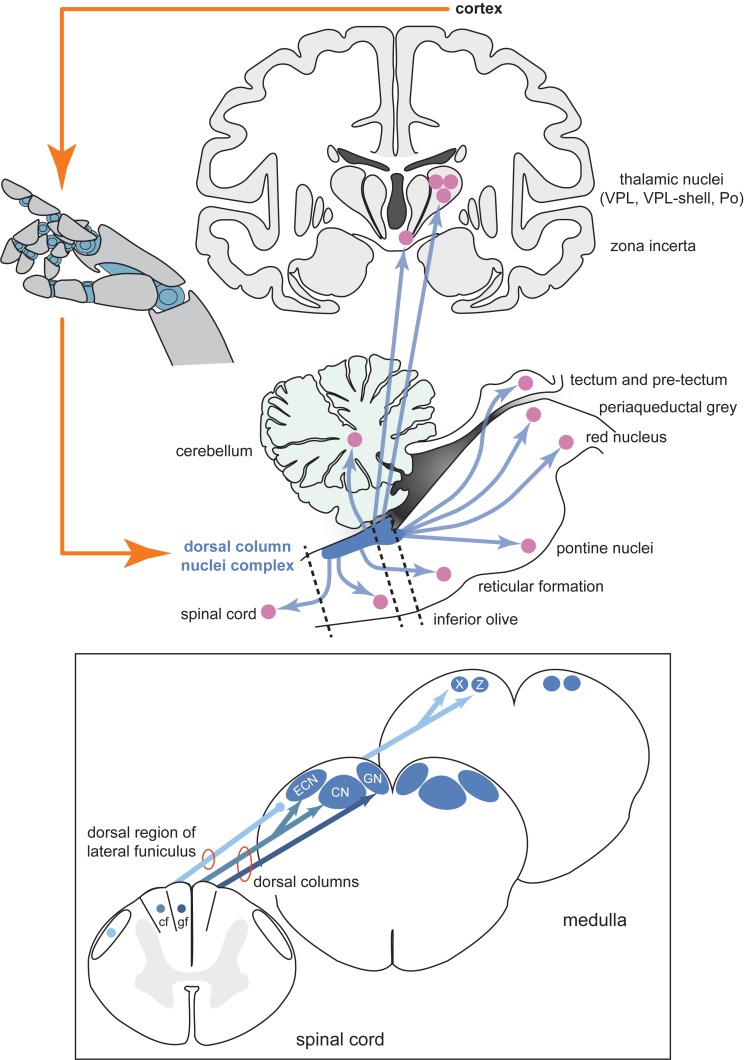
Schematic diagram of the inputs and projections of the dorsal column nuclei complex and information flow of a potential neural prosthesis with brainstem somatosensory feedback. Shown are schematic views of the forebrain (coronal section, top), hindbrain (parasagittal section, bottom), and spinal cord and medulla (transverse sections, insert). The dorsal column nuclei complex (DCNc; collectively: CN, GN, ECN, X and Z) projects to many sensorimotor targets in addition to the commonly described pathway through the ventroposterior lateral nucleus (VPL) of the thalamus. Providing sensory feedback by cortical stimulation bypasses these other essential targets involved in sensorimotor function. Compared to the cortical approach, a DCNc somatosensory neural prosthesis would provide a more realistic quality sensorimotor experience by accessing these other key sensorimotor regions. Dashed lines indicate spinal cord and brainstem cross-sections shown in the insert. Insert: The DCNc receives upper and lower body cutaneous and proprioception-related afferents via the cf and gf of the dorsal columns, respectively. Some lower body proprioception-related, and mixed modality upper and lower body afferents, travel to the DCNc via the dorsal region of the lateral funiculus including, but not limited to, the dorsal spinocerebellar tract. These afferents primarily synapse in X and Z. Therefore, to adequately restore all tactile and proprioceptive elements of somatosensation, the entire DCNc may require targeting. Abbreviations: cf, cuneate fasciculus; CN, cuneate nucleus; ECN, external cuneate nucleus; gf, gracile fasciculus; GN, gracile nucleus; Po, posterior group of the thalamus; VPL, ventroposterior lateral nucleus of the thalamus; X, nucleus X; Z, nucleus Z.

Spinal cord injury sufferers require somatosensory signals to be recreated in the central nervous system above the site of damage, so peripheral interfaces are not appropriate for this purpose. In our view, the dorsal column nuclei (DCN, comprising the gracile and cuneate nuclei) and its complex (DCNc, comprising the DCN, external cuneate nuclei and nuclei X and Z), may be an ideal alternative target to the cortex as they are easily accessible, being located in a supraspinal position in the dorsal aspect of the brainstem medulla (Figure 1), and are one of the first processing sites for ascending somatosensory information from the entire body (excluding the head). As the DCNc are lower in the somatosensory processing hierarchy, it may prove easier for the brain to interpret artificial DCNc activation as naturalistic stimuli, mirroring the success of peripheral nerve interfaces. Perhaps the most crucial feature is that the DCNc are part of a distribution network that accesses not only the somatosensory cortex for conscious perception, but also other key brain regions including the cerebellum, tectum, pretectum, inferior olive, red nucleus, pontine nuclei, zona incerta, reticular formation, periaqueductal gray, and the spinal cord (Figure 1; Loutit et al., 2019b). Direct parallel access to these centers from the DCNc provides a distinct advantage as a neural prosthesis site over primary somatosensory cortex, which would not have the same direct access to other key sensorimotor systems. Congruently, the DCN have received attention as a prospective somatosensory neural prosthetic target. Recently, it was shown that chronically implanted microelectrode arrays in monkeys can collect stable recordings, and variations in electrical DCN stimulation can elicit behavioral responses that demonstrate perceptual discrimination (Richardson et al., 2015, 2016; Sritharan et al., 2016; Suresh et al., 2017). While these studies establish the DCNc as a potential target, there is a severe lack of fundamental knowledge of the DCNc functional organization and somatosensory signal processing, which needs to be addressed before this region can be pursued as a feasible neural prosthetic target.

Here, we review recent work on the functional organization and somatosensory-evoked signals of key contributors to the DCNc. We suggest that the DCNc show promise as a target for a somatosensory neural prosthetic device. We discuss some of the potential limitations of the DCNc as a neural prosthetic target and propose future directions that are necessary before development of a DCNc neural prosthesis can begin.

## DCNc Functional Organization

Effective activation of the DCNc to elicit somatosensory percepts will require precise knowledge of its functional organization. The key components of the DCNc necessary to appreciate its potential use for neuroprosthetics are the gracile and cuneate nuclei, which are recipient of tactile (and other) inputs from lower and upper body afferents, respectively, and the external cuneate nuclei, nuclei X, and nuclei Z, which are key regions of proprioceptive inputs (summarized by Figure 1). For further details, we have recently performed a comprehensive review of the structural organization and the inputs and outputs of the DCNc (Loutit et al., 2019b). Interestingly, despite DCN neurons being somatotopically arranged across the coronal plane of the nuclei, evidence from our laboratory suggests that activity hotspots are spatially displaced across the surface of these nuclei when evoked from different stimulus locations (Loutit et al., 2017, 2019a). Recently, Suresh et al. (2017) also showed that macaque cuneate somatotopic maps are rostrocaudally organized, in addition to the medial-lateral and dorsal-ventral organization (Loutit et al., 2019b). Accordingly, attempts to stimulate cutaneous upper and lower body regions of the DCN, in addition to proprioceptive regions in the external cuneate nuclei, nuclei X, and nuclei Z, will have to target reasonably spatially displaced areas (Loutit et al., 2019b). Moreover, selective targeting of receptive fields to communicate contact location may require activation of neurons at different depths under the same surface region, which could prove difficult with current brain-machine-interface technologies. However, the different rostrocaudal sites could be exploited to activate neural populations with current multi-electrode array technologies that could otherwise not access different depths at adequate resolution under the same surface region.

Surprisingly, we also found that gracile activity hotspots evoked from bilateral nerve pairs were asymmetrically organized (Loutit et al., 2017, 2019a). This may indicate that the underlying structures that generate this activity are also asymmetrically organized. Previously, some variability in the somatotopic arrangement of hindlimbs has been shown in the gracile nuclei of cats and rats (Millar and Basbaum, 1975; Maslany et al., 1991). Little is known about lateralization in subcortical structures, but cortical lateralization related to handedness is a common phenomenon in mammals, including rats (Denenberg, 1981; Nudo et al., 1992; Dassonville et al., 1997; Hopkins and Cantalupo, 2004; Rogers, 2009). Recent evidence suggests that lateralization of cortical structures might, in part, result from gene expression asymmetries in the spinal cord (Ocklenburg et al., 2017). If this is the case, it is likely that the DCNc and other nuclei along the motor and sensory pathways between the spinal cord and the cortex will also show structural asymmetry. Preliminary data from our laboratory indicate that DCNc functional lateralization is related to paw dominance in the rat. DCNc asymmetries will need to be considered when designing a future DCNc neural prosthesis, but if each neural prosthesis is tailored to an individual, asymmetry is unlikely to be of major concern.

## DCNc Neurophysiology

To be a useful neural prosthetic target we propose that the DCNc neurophysiological characteristics must meet some preliminary conditions. Firstly, somatosensory-evoked DCNc signals must be shown to be robust and reproducible and, secondly, they must contain information that can be used to predict the location and quality of somatosensory stimuli. Signal features that reliably predict the location and quality of somatosensory stimuli indicate that they are relevant to the peripheral somatosensory event. Therefore, these features may inform the construction of artificial stimulus patterns that can activate DCNc neurons and elicit somatosensory percepts of natural quality.

Toward this goal, our laboratory has characterized somatosensory-evoked DCN surface activity (Loutit et al., 2017) and used feature-learnability (Loutit and Potas, 2019; Loutit et al., 2019a) – a machine-learning approach for evaluating the relevance of input features to the outputs – to determine the most useful signal features for predicting the location and quality of somatosensory stimuli. We consistently found DCN signal features contain a unique profile of high-frequency (HF) and low-frequency (LF) content when evoked from predominantly cutaneous nerves compared to nerves with mixed afferents from deep and cutaneous structures (Loutit et al., 2017, 2019a), and similarly, from tactile- or proprioceptive-dominated mechanical stimuli (Loutit and Potas, 2019). We extracted signal features from surface potential recordings of the DCN, and by using feature-learnability, were able to establish the relevance, or importance, of information inherently encoded in these signal features for (1) predicting the nerve or paw that was stimulated, and (2) the tactile or proprioceptive quality of the stimuli (Loutit and Potas, 2019; Loutit et al., 2019a). The best individual HF DCN signal features predicted electrically and mechanically evoked somatosensory events with 87 and 70% accuracy, respectively, while the best LF features achieved 90 and 66% accuracy, respectively (Loutit and Potas, 2019; Loutit et al., 2019a), suggesting that both frequency bands represent physiological events relevant to the somatosensory stimuli.

Before artificial stimulus features can be designed to activate the DCNc, we need a greater understanding of somatosensory information coding in the DCNc. In the following section we discuss how knowledge of DCN functional organization and signal features, such as those described above, can inform the development of a future neural prosthetic device.

## Discussion

### A Potential DCNc Neural Prosthesis

Two groups have successfully achieved chronic implantation of Utah microelectrode arrays (Blackrock Microsystems) and floating microelectrode arrays in the cuneate nuclei of macaques, which were able to obtain stable recordings up to about 140 days post-implantation, and awake behaving macaques could detect amplitude-dependent DCN stimulation at 100 Hz (Richardson et al., 2015, 2016; Sritharan et al., 2016; Suresh et al., 2017). These studies have demonstrated proof of principle that chronic microelectrode array implants are stable in this region and that peripheral receptive fields can be selectively activated. However, the next key advancement will be the careful selection and testing of parameters for DCN stimulation to elicit naturalistic sensations.

The HF and LF DCN activity we have investigated is either directly recorded volleys of action potentials arriving in the DCN from afferent fibers (HF activity), or from the subsequent activation of DCN neurons (HF and LF activity). Therefore, to ensure that neural activity giving rise to both the HF and LF features is restored, consideration should be given to whether the best approach is to activate the cuneate and gracile fasciculus fibers of the dorsal column (DC), rather than DCN neurons, or perhaps both. Some interesting recent studies show that rats and monkeys can detect differences in frequency and location of epidural DC stimulation (Yadav et al., 2019, 2020), which is a potential approach for restoring somatosensory feedback and is an FDA-approved method of chronic pain management in humans. However, exclusively stimulating the DC may limit the ability to activate lower body proprioceptive, and potentially other somatosensory information that travels in the lateral funiculus, projecting to nuclei X and Z (Loutit et al., 2019b). Like the sensorimotor cortex, the DC and the DCN are both somatotopically organized and modality segregated (Whitsel et al., 1969, 1970; Niu et al., 2013; Loutit et al., 2019b), making them useful targets for signaling contact location, and different sensory qualities. To restore tactile and proprioceptive sensation for both the upper and lower body, it will be necessary to either incorporate the lateral funiculus with DC stimulation, or target the entire DCNc.

The modular arrangement of the DCNc may be advantageous for neural prosthetic applications because each specific target relevant to the deficit region can be restored. The modularity and apparent sparsity of interconnectedness within the DCNc suggests that key regions can be specifically targeted with a neural prosthesis, without activating adjacent intact regions. Moreover, the entire body except the head is represented within a relatively small area across the DCNc surface (approximately 16 mm^2^), facilitating access to large body regions. One of the challenges faced in ICMS is the large cortical surface area dedicated to processing somatosensory information from the human hand (Collinger et al., 2018), which spans approximately 4 cm along the post-central gyrus (Flesher et al., 2016). Current microelectrode arrays are relatively small (typically 4 mm × 4 mm) and therefore can only evoke sensations in small regions of the hand. While the compactness of the DCNc is advantageous, a key challenge will be increasing the number and density of electrodes used to activate the DCNc with high precision, however, this challenge is currently met with intense research effort.

Compared to attempts to restore sensation in tetraplegics, approaches to restore somatosensation in amputees with upper limb prostheses have been relatively successful, and may guide approaches in the DCNc. Peripheral nerve stimulation has been successful in eliciting percepts of contact location, pressure, proprioceptive qualities, and textural discrimination (Dhillon and Horch, 2005; Clark et al., 2014; Tan et al., 2014, 2015; Oddo et al., 2016). Typical stimulation protocols deliver trains of electrical pulses to peripheral nerves with amplitudes varying between 20–300 μA, and frequencies of 10–300 Hz (Raspopovic et al., 2014; Oddo et al., 2016; Valle et al., 2018; George et al., 2019), which are similar to those used in cortical stimulation (Johnson et al., 2013; Flesher et al., 2016; Salas et al., 2018). When linearly encoded, a higher value from a robotic force sensor produces an increased stimulation frequency or amplitude, which induces perception of increased stimulus intensity (Johnson et al., 2013; Raspopovic et al., 2014; Flesher et al., 2016; George et al., 2019).

While these linear encoders can elicit perception of changes in stimulus intensity, they are often not perceived as naturalistic by the user. Of particular interest is trying to create biomimetic artificial touch, which would create naturalistic activation patterns, and therefore naturalistic sensations, in response to spatiotemporal stimulation patterns (Saal and Bensmaia, 2015). Biomimetic stimulus patterns mimic attributes of fast- or slowly adapting afferents, by modulating stimulus frequency or amplitude at different phases of a stimulus presentation e.g. varying the stimulus at the onset, offset, static, or dynamic phases of a stimulus. Indeed, spike timing and temporal features of spike trains, independent of mean spike rates, encode a variety of tactile stimulus features (Johansson and Birznieks, 2004; Saal et al., 2009; Birznieks et al., 2010; Birznieks and Vickery, 2017; Ng et al., 2018). Such parameters have facilitated the instantaneous estimation of fingertip forces, essential for tasks like object manipulation (Khamis et al., 2015). Recent biomimetic testing has shown that spatiotemporal stimulation patterning that mimics firing patterns of different fast- and slowly adapting peripheral afferents generates more natural percepts to the user (Oddo et al., 2016; Valle et al., 2018; George et al., 2019). This is complemented by evidence suggesting that vibration and intensity can be multiplexed by peripheral neural coding, without the need to alter current intensity (Ng et al., 2019). However, a biomimetic approach may be technologically limited by the number of electrodes that can be implanted in a peripheral nerve, to selectively activate individual or small groups of afferents of different submodalities.

Saal and Bensmaia (2015) have suggested that in higher centers, it may not be necessary to selectively activate so many neurons. Their reinterpretation of peripheral afferent coding suggests that all afferent classes encode aspects of most tactile features (Saal and Bensmaia, 2014), and electrophysiological evidence suggests massive cutaneous primary afferent convergence onto multiple DCN neurons (Witham and Baker, 2011; Bengtsson et al., 2013; Jörntell et al., 2014). Therefore, it may be possible to use fewer electrodes to biomimetically activate a small sample of neurons in the DCN that convey more complex, naturalistic, tactile features than attempting to stimulate a larger number of primary afferents.

One group has shown that microstimulation applied to the DCN of macaques at 100 Hz could be detected at amplitudes of 45–80 μA (Sritharan et al., 2016), which is comparable to cortical stimulation ranges that elicit somatosensory percepts. It is unclear what perceptual qualities are elicited from DCN stimulation as there are no reported studies of DCN electrical stimulation in humans. However, evidence from the abovementioned peripheral nerve studies suggests that future attempts to stimulate the DCN may benefit from adopting a biomimetic approach.

### Limitations

**FIGURE 2:**
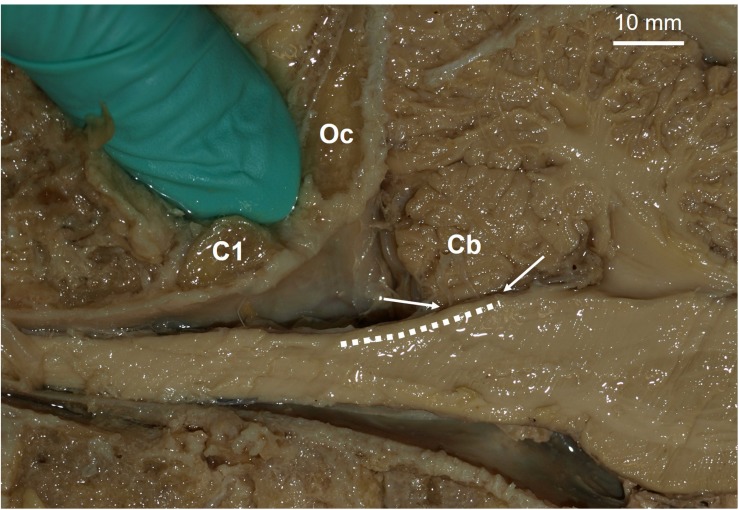
Proposed site for a potential dorsal column nuclei complex somatosensory neural prosthesis. Parasagittal view of a human brainstem and cerebellum (Cb). Dashed line indicates location of dorsal column nuclei complex (DCNc). As brain-machine-interface technology advances, future approaches may incorporate soft nanowire electrode “threads” that would permit stable targeted neural excitation during movement of the brainstem. Arrows indicate the DCNc region covered by the Cb, which can be easily retracted for electrode implantation if required. The gloved finger is inserted in the space proposed for surgical access, i.e. between the 1st cervical segment (C1) and the occipital bone (Oc).

Aside from the potential benefits, there are several concerns for targeting a somatosensory neural prosthesis in the DCNc. The required surgery to place electrodes in the DCNc is more invasive than in the cortex. Currently, the surgery will likely involve cutting the trapezius, splenius capitis, and semispinalis capitis muscles of the posterior neck, whereas cutting, removal, and replacement of a section of cranium is safer and routinely performed in humans. Moreover, the primary goal for spinal cord injury patients is to restore motor control. The state-of-the-art upper limb motor prostheses for tetraplegics are driven by neural activity recorded from electrodes in the motor cortex (Collinger et al., 2013; Ajiboye et al., 2017). A single surgery is required to place both motor and somatosensory arrays on the cortex to restore sensorimotor functions. Conversely, to achieve sensory feedback using a DCNc somatosensory neuroprosthesis will require two surgeries; one in the cortex and one in the brainstem (Figure 2 shows the proposed site). Future investigations will need to assess these risks and demonstrate that the sensory improvements achieved by a DCNc implant outweighs that which can be achieved by a cortical implant.

Current chronic DCNc electrode arrays risk being moved or damaged by head and neck movements. Several failed experiments in macaques have been reported due to damaging the wire bundles that transmit electrical signals between the electrode arrays and headstages fixed to the skull, or from the arrays falling out (Suresh et al., 2017). Thus far, rigid microelectrode arrays have been used, but the development of new technologies that use less rigid array structures to accommodate movement, could solve this issue. For example, recently an approach has been developed that delivers small flexible electrode “threads” into the brain (Musk, 2019). Each thread can be targeted with micrometer precision and each array can have up to 3,072 active electrodes. New technologies such as this would permit the insertion of a network of flexible electrodes that could be sewn in place at high resolution throughout the entire DCNc (Figure 2), while permitting stable recordings during movement of brain tissues and without causing tissue damage.

The safety and efficacy of DCNc array insertion and electrical stimulation is also yet to be established. As described above, experiments in macaques showed that DCNc stimulation could be detected with currents in the range deemed safe to avoid neural damage (<100 μA per electrode; 20 μC/phase) (Chen et al., 2014; Rajan et al., 2015; Flesher et al., 2016). However, DCNc tissues will need to be analyzed following chronic microelectrode implantation and stimulation, to determine if the effects differ to that shown in the cortex. Moreover, penetrating electrodes and electrical stimulation in the DCNc pose a risk of damaging or activating neurons in respiratory control centers including the rostral ventrolateral medulla, the ventral respiratory column, and the nucleus of the solitary tract (Zoccal et al., 2014). While the ventral position of the first two centers are unlikely to be affected by DCNc stimulation, there is some risk of physically penetrating or activating the nucleus of the solitary tract, which is located near the DCNc ventral border. In our laboratory we have routinely inserted electrode arrays in the gracile and cuneate nuclei of rats that occasionally penetrate the ventral border without affecting any cardiorespiratory functions. The two macaque studies using chronic arrays in the DCNc also reported no issues with cardiorespiratory function either from physical penetration or from stimulation up to 100 μA (Richardson et al., 2016; Sritharan et al., 2016; Suresh et al., 2017). Nevertheless, the potential to cause adverse effects on respiratory control or coupling of cardiovascular and respiratory activities is a serious concern, and safe stimulation levels and penetration depths will need to be established.

Finally, spinal cord or peripheral nerve injury has been shown to cause changes in DCN somatotopy and is thought to be a crucial driver of some cortical somatotopic reorganization (Kambi et al., 2014). This will need to be considered in each subject, to determine how best to target microelectrode arrays.

## Future Directions

We believe that a DCNc somatosensory neural prosthesis is a goal worth pursuing and may provide advantages over cortical somatosensory neural prostheses. However, there are a number of concerns that need be addressed, regarding the safety and efficacy of placing microelectrode arrays and electrically stimulating in the DCNc, before a brainstem somatosensory neural prosthesis can be considered feasible. Compared to peripheral nerves and the somatosensory cortex, there is also a dire lack of knowledge about how somatosensory information is coded in the DCNc, which demands future efforts directed toward the understanding of how tactile and proprioceptive features are represented in DCNc neurons. The next frontier will then be to determine how to implement neural codes using a biomimetic approach to artificially stimulate the DCNc which is already connected to multiple sensorimotor systems, including conscious (likely involving the cortex) and unconscious (non-cortical) pathways. Such an approach could enable the subject to receive tactile and proprioceptive sensations from an anthropomorphic robotic limb for complete sensorimotor integration into multiple systems.

## Author Contributions

Both authors wrote the manuscript.

## Conflict of Interest

The authors declare that the research was conducted in the absence of any commercial or financial relationships that could be construed as a potential conflict of interest.
